# Complexed Crystal Structure of the Dihydroorotase Domain of Human CAD Protein with the Anticancer Drug 5-Fluorouracil

**DOI:** 10.3390/biom13010149

**Published:** 2023-01-11

**Authors:** En-Shyh Lin, Yen-Hua Huang, Po-Chun Yang, Wei-Feng Peng, Cheng-Yang Huang

**Affiliations:** 1 Department of Beauty Science, National Taichung University of Science and Technology, Taichung City 403, Taiwan; 2 Department of Biomedical Sciences, Chung Shan Medical University, Taichung City 402, Taiwan; 3 Department of Medicine, College of Medicine, Chung Shan Medical University, Taichung City 402, Taiwan; 4 Department of Pediatrics, National Taiwan University Children’s Hospital, Taipei 100, Taiwan; 5 Department of Medical Research, Chung Shan Medical University Hospital, Taichung City 402, Taiwan

**Keywords:** dihydroorotase, CAD, 5-fluorouracil, anticancer drug, crystal structure, pyrimidine biosynthesis, dihydropyrimidinase, fluorescence quenching, dynamic loop

## Abstract

Dihydroorotase (DHOase) is the third enzyme in the pathway used for the biosynthesis of pyrimidine nucleotides. In mammals, DHOase is active in a trifunctional enzyme, CAD, which also carries out the activities of carbamoyl phosphate synthetase and aspartate transcarbamoylase. Prior to this study, it was unknown whether the FDA-approved clinical drug 5-fluorouracil (5-FU), which is used as an anticancer therapy, could bind to the DHOase domain of human CAD (huDHOase). Here, we identified huDHOase as a new 5-FU binding protein, thereby extending the 5-FU interactome to this human enzyme. In order to investigate where 5-FU binds to huDHOase, we solved the complexed crystal structure at 1.97 Å (PDB ID 8GVZ). The structure of huDHOase complexed with malate was also determined for the sake of comparison (PDB ID 8GW0). These two nonsubstrate ligands were bound at the active site of huDHOase. It was previously established that the substrate *N*-carbamoyl-L-aspartate is either bound to or moves away from the active site, but it is the loop that is extended towards (loop-in mode) or moved away (loop-out mode) from the active site. DHOase also binds to nonsubstrate ligands via the loop-out mode. In contrast to the *Escherichia coli* DHOase model, our complexed structures revealed that huDHOase binds to either 5-FU or malate via the loop-in mode. We further characterized the binding of 5-FU to huDHOase using site-directed mutagenesis and the fluorescence quenching method. Considering the loop-in mode, the dynamic loop in huDHOase should be a suitable drug-targeting site for further designing inhibitors and clinical chemotherapies to suppress pyrimidine biosynthesis in cancer cell lines.

## 1. Introduction

5-Fluorouracil (5-FU) is an FDA-approved drug that is used to target the enzyme thymidylate synthase (TSase) for anticancer chemotherapy [1,2,3]. 5-FU induces cytotoxicity by inhibiting the action of TSase or by causing RNA miscoding. Although many new drugs have been developed, 5-FU is still one of the most commonly used chemotherapeutic agents for the systemic treatment of colorectal, breast, head, and neck cancers [4]. More than a dozen different proteins are known to bind to 5-FU, including dihydropyrimidinase (DHPase) [5]. 5-FU-associated toxicity was reported in asymptomatic patients with DHPase deficiency who underwent anticancer therapy [6]. These patients suffered from severe toxicity, including death, after treatment with 5-FU [7]. In addition, microbiota can also regulate and modulate the host response to 5-FU [2,8]. For example, the active gut microbiota capable of producing bromovinyluracil can increase the systematic concentrations of 5-FU and caused the death of 16 patients in Japan [8,9]. Thus, the whole interactome of 5-FU should be built for detailed clinical pharmacokinetic and toxicity analyses [10].

DHPase [11,12], dihydroorotase (DHOase) [13], allantoinase (ALLase) [14], hydantoinase (HYDase) [15,16], and imidase [17,18] are members of the cyclic amidohydrolase family [11]. These metal-dependent enzymes catalyze the hydrolysis of the cyclic amide bond of each substrate in 5- or 6-membered rings in the metabolism of purines and pyrimidines [19]. Almost all of these enzymes contain a similar binuclear metal center consisting of four His, one Asp, and one post-translational carbamylated Lys (Kcx) residue [11]. These cyclic amidohydrolases have a similar active site and mechanism for catalysis, but their substrate selectivity and specificity are different [20]. Given that DHPase can form a stable complex with 5-FU, it is fascinating to investigate whether other cyclic amidohydrolases, e.g., the DHOase studied here, are also capable of binding to 5-FU.

**Figure 1:**
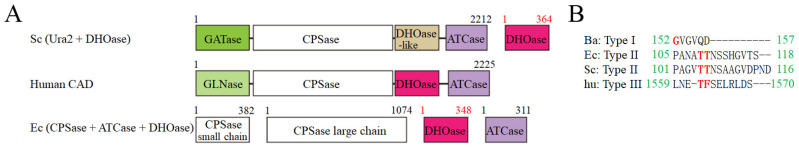
Comparison of DHOases. (**A**) The gene products for the first three reactions of pyrimidine biosynthesis are different among species. Human CAD consists of DHOase, CPSase, and ATCase domains fused covalently. *E. coli* (Ec) CPSase, DHOase, and ATCase function separately. *S. cerevisiae* (Sc) CPSase, and ATCase activities are present in a single bifunctional protein, Ura2. Ura2 is a CAD-like polypeptide that contains a defective DHOase-like domain. (**B**) Sequence alignment of the flexible loop. The amino acids that are involved in catalysis are in red. The sequence composition and the length of these flexible loops are significantly distinct.

DHOase catalyzes the cyclization of *N*-carbamoyl-L-aspartate (CA-asp) to L-dihydroorotate (DHO) in the third step of de novo pyrimidine biosynthesis [21]. DHOase activity is found in all organisms for the biosynthesis of pyrimidine nucleotides, but phylogenetic and structural analyses revealed at least three different DHOase forms (Figure 1A) [21,22]. *Bacillus anthracis* DHOase (BaDHOase) [23] and *Aquifex aeolicus* DHOase (AaDHOase) [24] are type I DHOases (approximately 45 kDa), which are evolutionarily ancient and larger than their type II counterparts (approximately 38 kDa), such as those from most eubacteria, fungi, and plants. A structural analysis recently indicated that human DHOase (huDHOase) should be reclassified from type I DHOase to type III DHOase [22]. In mammals, DHOase is a part of a single trifunctional polypeptide of 240 kDa, namely carbamoyl phosphate synthetase (CPSase)/aspartate transcarbamoylase (ATCase)/DHOase protein (CAD), that selfassembles into a hexamer of 1.5 MDa [25]. In yeasts, CPSase and ATCase are present in a single bifunctional protein, Ura2, which is a CAD-like polypeptide that contains a defective DHOase-like domain [26]. In most prokaryotic organisms, CPSase, ATCase, and DHOase are expressed separately and function independently [27]. We previously found that the type II DHOase from *Saccharomyces cerevisiae* (ScDHOase) can bind to 5-FU [28]. Prior to this study, it was unknown whether the type I and type III DHOase could interact with 5-FU, as is the case for the type II enzyme.

All known DHOases can exist as a monomer or dimer. The type II DHOase from *Escherichia coli* (EcDHOase) was the first to have its structure determined [13], and EcDHOase functions as a dimer [27]. The complex crystal structure of the dimeric EcDHOase showed that the substrate CA-asp and the product DHO were found at different active sites [13]. A further structural study [29] of EcDHOase indicated that a flexible, dynamic loop is extended towards the active site or moved away from the active site when CA-asp is either bound to (loop-in mode) or moved away from the active site (loop-out mode). In addition, EcDHOase can bind to the inhibitor 5-fluoroorotate (5-FOA) via the loop-out mode [29]. Mutational analyses indicated that the two residues, T109 and T110 (Figure 1B), on the flexible loop are important to stabilize the transition state during the whole catalytic cycle of EcDHOase [30]. Similarly, huDHOase can also bind to these nonsubstrate ligands via the loop-out mode [22]. However, recent structural data reveal that ScDHOase binds to nonsubstrate ligands, such as malate [31], 5-FU [28], 5-FOA [32], and plumbagin [33], via the loop-in mode. Thus, the idea that the loop movement mechanisms for the catalytic reactions of EcDHOase and huDHOase are species-dependent should be reproposed.

In this study, we identified huDHOase as a novel 5-FU binding protein, thereby extending the 5-FU interactome to this human enzyme. In contrast to the proposed model of EcDHOase, huDHOase is bound to these nonsubstrate ligands via the loop-in mode. In addition, mutational and structural analyses indicate that the binding modes of the nonsubstrate ligands of huDHOase also differed from ScDHOase and *Pseudomonas aeruginosa* DHPase (PaDHPase). When considering the loop-in mode revealed by our complexed structures of huDHOase, the dynamic loop in DHOase should be a suitable drug-targeting site for inhibiting pyrimidine biosynthesis in order to suppress cancer-signaling pathways [34,35,36].

## 2. Materials and Methods

### 2.1. Protein Expression and Purification

The expression vector pET21b-huDHOase [33,37] was transformed into *E. coli* BL21 (DE3) cells and grown in LB medium at 37 °C. The overexpression was induced by incubating with 1 mM isopropyl thiogalactopyranoside for 9 h. Recombinant huDHOase (the amino acid residues 1456–1846 in human CAD protein) containing the *C*-terminal His tag was purified from the supernatant by using Ni^2+^-affinity chromatography. The recombinant protein was eluted with a linear imidazole gradient and dialyzed against a dialysis buffer (20 mM Tris-HCl and 0.1 M NaCl, pH 7.9; Buffer A). The protein purity was >97%, as determined using SDS-PAGE.

### 2.2. Site-Directed Mutagenesis

The huDHOase mutants were generated according to the QuikChange site-directed mutagenesis kit protocol (Stratagene; LaJolla, CA, USA) by using the wild-type plasmid pET21b-huDHOase as a template. The presence of the mutation was verified by DNA sequencing in each construct. The recombinant mutant proteins were purified using the protocol for the wild-type huDHOase by Ni^2+^-affinity chromatography.

### 2.3. Crystallization Experiments

Before crystallization, the purified huDHOase was concentrated at 20 mg/mL in Buffer A. The crystals of the huDHOase–malate complex were grown at room temperature through hanging drop vapor diffusion in 0.1 M malate and 1.6 M potassium sodium tartrate tetrahydrate at pH 6.0. For the huDHOase-5-FU complex, the complexed crystals were grown in 0.1 M MES, 1.6 M potassium sodium tartrate tetrahydrate, and 200 μM 5-FU at pH 6.5. These crystals reached full size in 7–13 days. The crystals were transferred from a crystallization drop into the cryoprotectant solution (2 μL) with precipitant solution containing glycerol (25–30%) for a few seconds, then mounted on a synthetic nylon loop (0.1–0.2 mm), flash cooled in liquid N_2_, and analyzed in the beamline 07A1 of the National Synchrotron Radiation Research Center (NSRRC; Hsinchu, Taiwan).

### 2.4. X-ray Diffraction Data and Structure Determination

Data were collected using an EIGER2 X 16M Detector at an SPXF beamline TPS 07A at NSRRC (Taiwan). Data sets were indexed, integrated, and scaled by HKL-2000 [38] and XDS [39]. Phasing, density modification, and model building were performed using the AutoSol program [40] in the PHENIX [41]. The iterative model building and structure refinement were performed using Refmac in the CCP4 software suite [42] and phenix.refine in the PHENIX software suite. Phasing of huDHOase complexed with malate or 5-FU was determined through the molecular replacement software Phaser MR [43] by using huDHOase (PDB ID 4C6C) as a search model. The correctness of the stereochemistry of the models was verified using MolProbity [44]. Atomic coordinates and related structure factors were deposited in the PDB with accession codes 8GW0 (the huDHOase–malate complex) and 8GVZ (the huDHOase–5-FU complex).

### 2.5. Determination of the Dissociation Constant (K_d_)

The *K*_d_ value of purified huDHOase was determined using the fluorescence quenching method, as previously described for the DHOase and DHPase [20,45,46]. Briefly, an aliquot of the compound was added into the solution containing huDHOase (1 μM) and 50 mM HEPES at pH 7.0. The decrease in the intrinsic fluorescence of DHOase was measured at 340 nm upon excitation at 280 nm and 25 °C with a spectrofluorometer (Hitachi F-2700; Hitachi High-Technologies, Tokyo, Japan). The *K*_d_ was obtained using the following equation: Δ*F* = Δ*F*_max_ − *K*_d_(Δ*F*/[5-FU]).

## 3. Results

### 3.1. Crystallization of huDHOase in Complex with Malate and 5-FU

Prior to this study, the complexed crystal structure of huDHOase with either malate or 5-FU was not available. In order to understand whether binding occurs at the active site and investigate the binding mode, the structural information of these huDHOase complexes is needed. As a first step toward determining the binding mode of huDHOase by malate or 5-FU, we attempted to cocrystallize these complexes to further obtain the crystal structures. Recombinant huDHOase (the amino acid residues 1456–1846 in human CAD protein) was overexpressed in E. coli and purified by Ni^2+^-affinity chromatography. Through crystallization screening, the crystals of the huDHOase–malate complex were grown at room temperature through hanging drop vapor diffusion in 0.1 M malate and 1.6 M potassium sodium tartrate tetrahydrate at pH 6.0. For the huDHOase–5-FU complex, the crystals were grown in 0.1 M MES, 1.6 M potassium sodium tartrate tetrahydrate, and 200 μM of 5-FU at pH 6.5.

### 3.2. Overall Structure of the huDHOase Complexes

**Figure 2:**
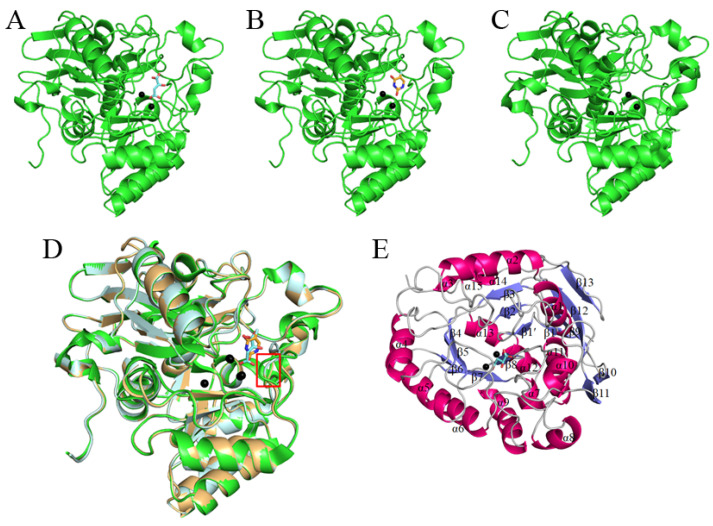
Structures of huDHOase. (**A**) Ribbon diagram of huDHOase monomer complexed with malate. The two zinc ions in the active site are presented as black spheres. A malate molecule is shown in aquamarine. (**B**) Ribbon diagram of huDHOase complexed with the anticancer drug 5-FU. Two zinc ions were found in the active site of huDHOase. A 5-FU molecule is shown in light orange. (**C**) Structure of the apo-huDHOase. Three zinc ions were found in the active site of huDHOase. In comparison with these huDHOase structures, the binding of malate or 5-FU does not influence the overall structure of huDHOase. (**D**) The superimposed structures. Superimposing the apo (green), the malate-complexed (pale cyan), and the 5-FU-complexed (light orange) structures of huDHOase indicated the different positions of the active site loop (boxed in red). (**E**) Ribbon diagram of the huDHOase–malate complex with the secondary structures labeled. The global architecture of these huDHOase complexes revealed a TIM-barrel structure and consisted of 15 α-helices and 13 β-sheets.

The crystals of the huDHOase complexes belong to space group C222_1_, with one molecule per asymmetric unit (Table 1). The phases were obtained by molecular replacement using the structure of the apo-huDHOase (PDB ID 4C6C) as the search model [22]. The crystal structure of huDHOase complexed with malate (PDB ID 8GW0) and 5-FU (PDB ID 8GVZ) was solved at a 1.64 and 1.97 Å resolution, respectively. The amino acid residues 1456–1459 (the *N*-terminus) and 1822–1846 (the *C*-terminus) in the ternary structure of huDHOase were disordered and unobserved. The binding of malate (Figure 2A) or 5-FU (Figure 2B) did not significantly influence the overall structure of huDHOase (Figure 2C). Superimposing these three structures indicated the different positions of the active site loop (Figure 2D). Similar to the apo form (Figure 2C), the global architecture of these huDHOase complexes revealed a TIM-barrel structure that consisted of 15 α-helices and 13 β-strands (Figure 2E). The catalytic metal center in these huDHOase complexes consisted of His1471, His1473, His1590, His1614, and Asp1686, which were selfassembled. Lys1556 remained carbamylated (Kcx1556) regardless of ligand binding. However, these huDHOase complexes only contained two Zn ions, rather than the three Zn ions found in apo-huDHOase.

### 3.3. Potential Monomer–Monomer Interface of the huDHOase Complexes

**Figure 3:**
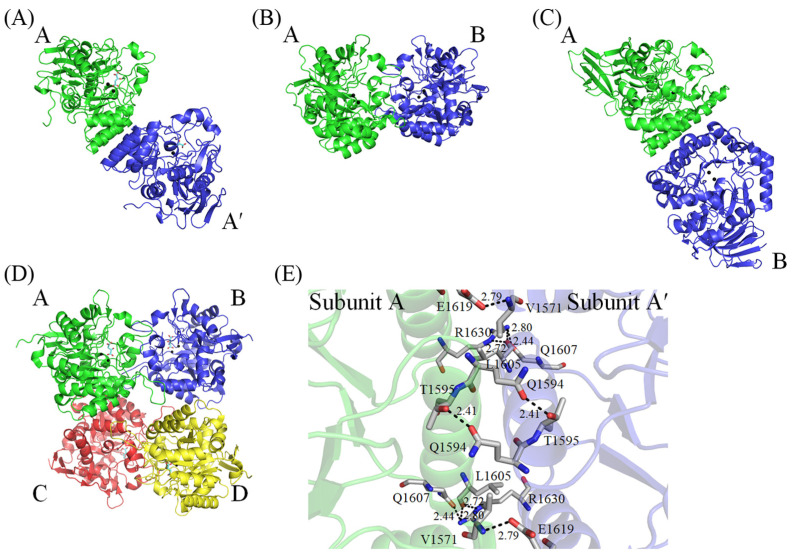
Homo-oligomerization in DHOases. (**A**) Ribbon diagram of the huDHOase dimer complexed with malate (PDB ID 8GW0). The dimerization mode of the huDHOase–5-FU complex is nearly identical to the huDHOase–malate complex and is therefore not shown. Each huDHOase monomer is color-coded. The two zinc ions in the active site are presented as black spheres. (**B**) Structure of the type II enzyme EcDHOase (PDB ID 2EG6). (**C**) Structure of the type I enzyme BaDHOase (PDB ID 3MPG). (**D**) Structure of a tetrameric ScDHOase (PDB ID 6L0A). (**E**) The formation of hydrogen bonds at the crystallographic-related monomer A–monomer A’ interface of the huDHOase–malate complex. The distance (Å) of the residues is shown. These H bonds further stabilized the dimerization core of the huDHOase complex.

**Figure 4:**
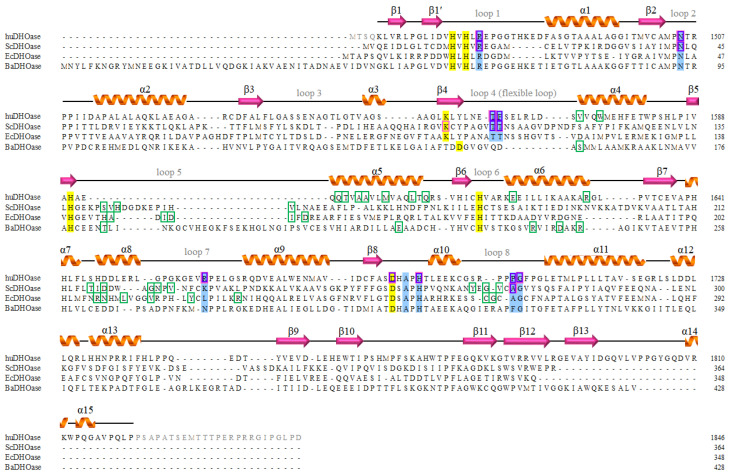
Multiple amino acid sequence alignment of DHOases. Sequences of huDHOase, ScDHOase, EcDHOase, and BaDHOase are analyzed and compared. huDHOase shares an identity with ScDHOase, EcDHOase, and BaDHOase by 39%, 21%, and 32%, respectively. The secondary structures of the huDHOase–malate complex are labeled. The unobserved residues 1456–1459 and 1822–1846 in the huDHOase ternary structure are colored in grey. The metal-binding sites are shaded in yellow. The substrate-binding sites are shaded in blue. The amino acids that are involved in the monomer–monomer interface via hydrogen bonding are boxed in green. The malate and 5-FU binding sites are boxed in purple and pink, respectively.

**Figure 5:**
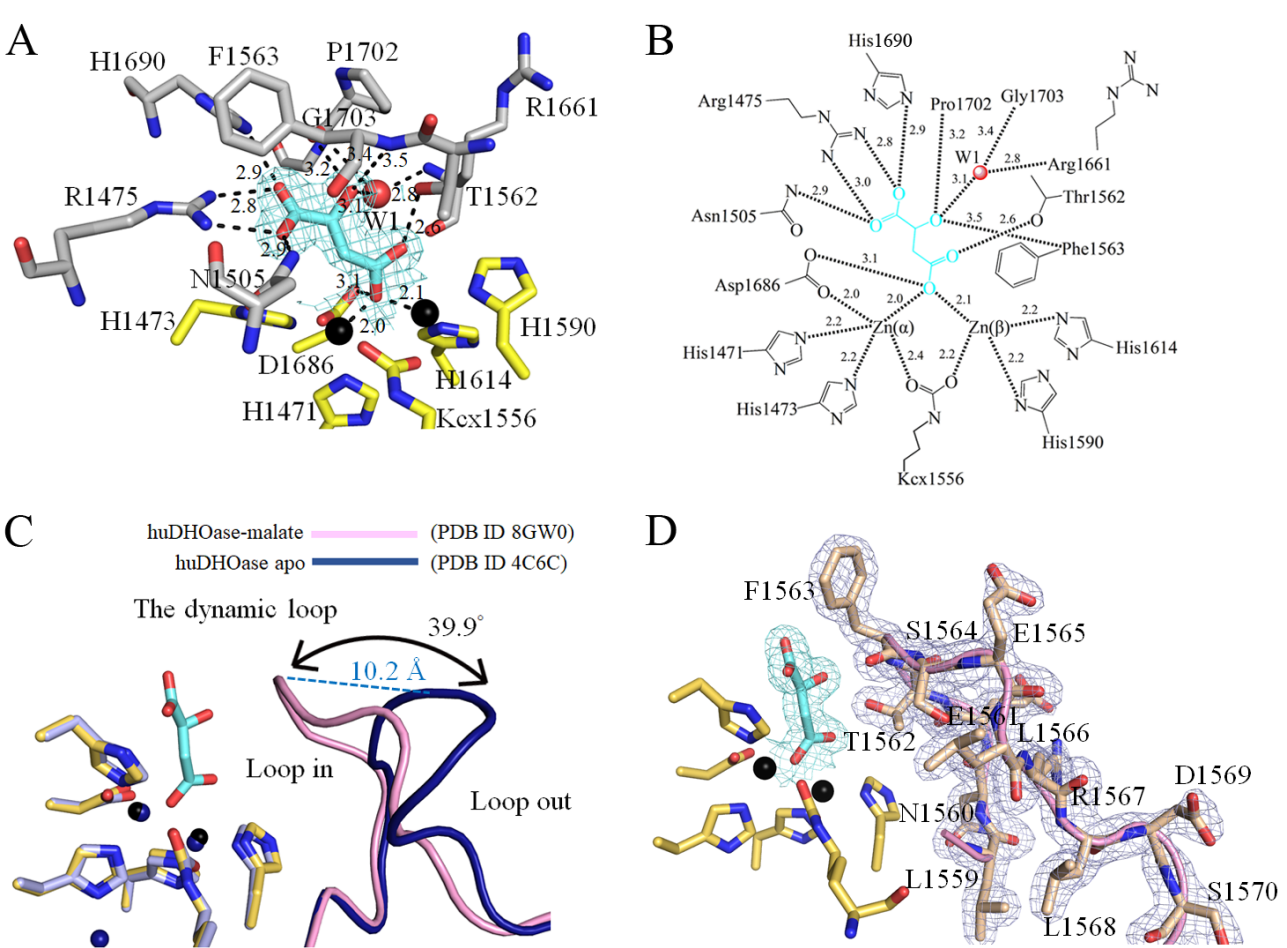
Malate binding mode. (**A**) The active site of huDHOase with malate. The composite omit map (aquamarine mesh, contoured at 1 σ) indicated the presence of malate in the active site of huDHOase. The two metal ions and residues R1475, N1505, T1562, F1563, R1661, D1686, H1690, P1702 and G1703 were involved in malate binding. Residues required for metal binding are colored in yellow. (**B**) The binding mode of malate. (**C**) Superposition of the apo-huDHOase and the huDHOase–malate complex. The structure revealed that huDHOase bound malate via the loop-in mode. As compared with the structure of the apo-huDHOase, the dynamic loop in the huDHOase–malate complex (pink) was shifted by a distance of 10.2 Å and an angle of 39.9^o^ for malate binding. Prior to this study, huDHOase bound nonsubstrate ligand via the loop-out mode, e.g., the dynamic loop (dark blue) did not interact with the ligand or with the rest of the active site of huDHOase. (**D**) The electron density of the residues on the dynamic loop in this structure was well-defined.

Given that all known DHOases can exist as a monomer or dimer, we analyzed and compared the monomer–monomer interface of the huDHOase–malate complex (Figure 3A), EcDHOase (Figure 3B), BaDHOase (Figure 3C), and ScDHOase (Figure 3D) to assess whether their dimer formation mechanisms were different. huDHOase shares an identity with EcDHOase, BaDHOase, and ScDHOase by 21%, 32%, and 39%, respectively. Although the crystals of our huDHOase complexes contained only one huDHOase molecule per asymmetric unit, huDHOase can form a dimer in a solution [22,31]. Accordingly, the crystallographic-related dimer A–A′ was selected through PISA [47] for this comparison. The monomer A and the crystallographic-related monomer A′ of the huDHOase complex with malate (Figure 3A) and 5-FU (data not shown; nearly identical to the huDHOase–malate complex) were interconnected through many hydrogen bonds and salt bridges (Figure 3E). These H bonds further stabilized the dimerization core of the huDHOase–malate and –5-FU complexes (Table 2). These bonds (<3 Å) included T1595(A)–Q1594(A′), R1630(A)–Q1607(A′), V1571(A)–E1619(A′), Q1594(A)–T1595(A′), Q1607(A)–R1630(A′), and E1619(A)–V1571(A′). The interactive residues (boxed in green) were almost different among these DHOases (Figure 4). Given that the critical residues for the dimerization of these huDHOase complexes were not conserved in EcDHOase, BaDHOase, and ScDHOase (Figure 4), we concluded that their dimer formation mechanisms are different [31].

### 3.4. Malate Binding Mode of huDHOase

Malate is an inhibitor of huDHOase [33]. In order to determine how malate can bind to huDHOase, we determined the crystal structure of huDHOase complexed with malate (Figure 5). This complexed structure revealed that malate is bound at the active site of huDHOase (Figure 5A). The two metal ions and residues, R1475, N1505, T1562, F1563, R1661, D1686, H1690, P1702, and G1703, were involved in malate binding (Figure 5B). R1475, N1505, and T1562 are also known as substrate-binding residues in huDHOase. Interestingly, huDHOase bound to malate via the loop-in mode (Figure 5C,D). Prior to this study, it was well-established that huDHOase binds to nonsubstrate ligands via the loop-out mode [22], e.g., the dynamic loop did not interact with the ligand or with the rest of the active site of huDHOase. In order to further analyze how this nonsubstrate ligand can bind to huDHOase via the loop-in mode, the structures of apo-huDHOase and the huDHOase–malate complex were superimposed (Figure 5C) for the sake of comparison. We found that the dynamic loop in the huDHOase–malate complex was shifted by a distance of 10.2 Å and an angle of 39.9^o^ for malate binding. In addition, the two residues on this dynamic loop in huDHOase that are crucial for catalysis, T1562 and F1563 [48], also interacted with malate (Figure 5B). Thus, we concluded that huDHOase can bind to malate via the loop-in mode, e.g., the flexible loop does not move away from the active site.

### 3.5. 5-FU Binding Mode of huDHOase

**Figure 6:**
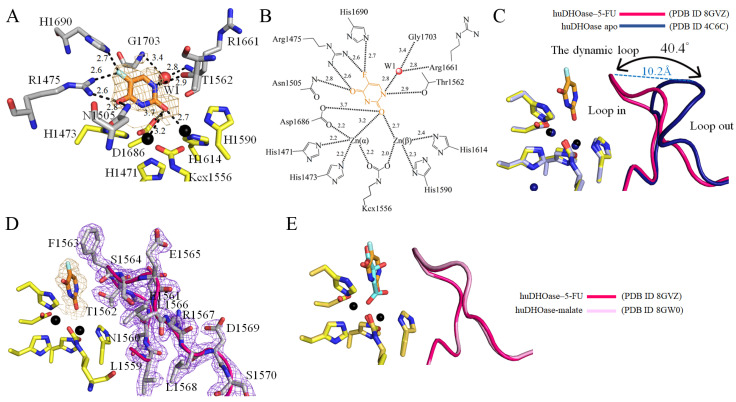
5-FU binding mode. (**A**) The active site of huDHOase with 5-FU. The composite omit map (orange mesh, contoured at 1 σ) indicated the presence of 5-FU in the active site of huDHOase. The two metal ions and residues R1475, N1505, T1562, R1661, D1686, H1690, and G1703 were involved in malate binding. Residues required for metal binding are colored yellow. (**B**) The binding mode of malate. (**C**) Superposition of the apo-huDHOase and the huDHOase–5-FU complex. The structure revealed that huDHOase bound 5-FU via the loop-in mode. As compared with the structure of the apo-huDHOase, the dynamic loop in the huDHOase–malate complex (red) was shifted by a distance of 10.2 Å and an angle of 40.4^o^ for 5-FU binding. Prior to this study, huDHOase bound nonsubstrate ligand via the loop-out mode, i.e., the dynamic loop (dark blue) did not interact with the ligand or with the rest of the active site of huDHOase. (**D**) The electron density of the residues on the dynamic loop in this structure was well-defined. (**E**) Superposition of the huDHOase–malate and the huDHOase–5-FU complexes. Their loop positions were almost identical.

DHOase is a key enzyme involved in pyrimidine biosynthesis. Prior to this study, it was unknown whether the FDA-approved clinical drug 5-FU, which is the best-known pyrimidine derivative for anticancer therapy, could bind to huDHOase. The complexed crystal structure of huDHOase with 5-FU was therefore ascertained in order to determine where the binding occurred and investigate the binding mode (Figure 6). The electron density of 5-FU was well-defined (Figure 6A). The orientation of 5-FU was easy to distinguish based on the location of the substituent. Similar to the huDHOase–malate complex, this complexed structure revealed that 5-FU is also bound at the active site of huDHOase (Figure 6A). However, this lacks some specific interactions compared to these two complexes. The two metal ions and residues, R1475, N1505, T1562, R1661, D1686, H1690, and G1703, were involved in 5-FU binding (Figure 6B); that is, F1563 and P1702 were involved in the binding of malate, but not 5-FU. Similar to the malate binding, 5-FU can bind to huDHOase via the loop-in mode (Figure 6C–E). Their loop positions were almost identical. When compared with apo-huDHOase, the dynamic loop in the huDHOase–5-FU complex was shifted by a distance of 10.2 Å and an angle of 40.4^o^ for 5-FU binding (Figure 6C). Accordingly, we concluded that huDHOase could bind to a nonsubstrate ligand, 5-FU, via the loop-in mode. Even though 5-FU is a nonsubstrate ligand for huDHOase, the flexible loop still did not move away from the active site.

### 3.6. Structure-Based Mutational Analysis

**Figure 7:**
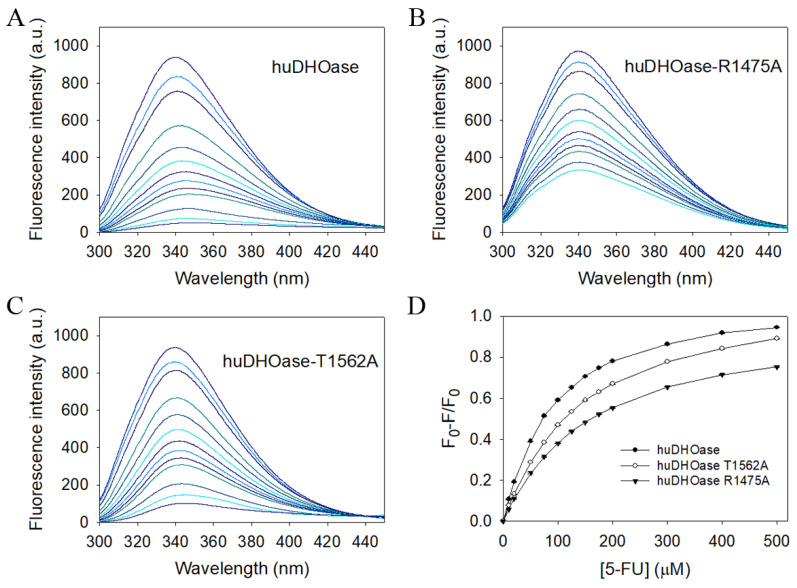
Fluorescence titration of huDHOase with 5-FU. (**A**) The fluorescence emission spectra of huDHOase with 5-FU of different concentrations (0–500 μM; 0, 10, 20, 50, 75, 100, 125, 150, 175, 200, 300, 400, and 500 μM). The decrease in intrinsic fluorescence of protein was measured at 340 nm upon excitation at 280 nm with a spectrofluorometer. The fluorescence intensity emission spectra of huDHOase were significantly quenched by 5-FU. (**B**) The fluorescence emission spectra of huDHOase-R1475A with 5-FU of different concentrations (0–500 μM). (**C**) The fluorescence emission spectra of huDHOase-T1562A with 5-FU of different concentrations (0–500 μM). (**D**) The titration curves for determining the *K*_d_ values. The *K*_d_ was obtained by the equation: Δ*F* = Δ*F*_max_ − *K*_d_(Δ*F*/[5-FU]). Data points are an average of 2–3 determinations within a 10% error.

Fluorescence quenching was performed in order to confirm the strength of the interaction of huDHOase with 5-FU and determine the *K*_d_ value (Table 3). Quenching refers to the complex formation process that decreases the fluorescence intensity of the protein. huDHOase displayed strong intrinsic fluorescence with a peak wavelength of 340 nm when excited at 280 nm. When different concentrations of 5-FU were individually titrated into the huDHOase solution, the intrinsic fluorescence was progressively quenched (Figure 7A). A total of 500 μM of 5-FU quenched the intrinsic fluorescence of huDHOase by 94.5%. Adding 5-FU caused a red shift (~ 8.5 nm; *λ*_max_ from 340 nm to 349 nm) in the huDHOase emission wavelength. Based on this observation, 5-FU was capable of forming a stable complex with huDHOase. As determined through the titration curve, the *K*_d_ value of huDHOase bound to 5-FU was 91.2 ± 1.7 μM (Table 3).

The complexed structure revealed huDHOase residues R1475 and T1562 as the major 5-FU binding sites (Figure 6), and 5-FU interacts with the side chains of these two residues. In order to investigate the contribution of these residues to 5-FU binding, alanine substitution mutants (Table 4) were constructed and analyzed by fluorescence quenching. These mutant proteins were purified using the same protocol for the wild-type huDHOase. We found that 500 μM of 5-FU quenched the intrinsic fluorescence of the mutant huDHOase-T1562A (Figure 7B) and huDHOase-R1475A (Figure 7C) by 89.0% and 75.3%, respectively. The *K*_d_ values of T1562A and R1475A bound to 5-FU were reduced to 146.5 ± 2.1 and 161.5 ± 1.6 μM, respectively. Accordingly, the interactions of 5-FU with these huDHOase residues (R1475 and T1562) were experimentally confirmed (Figure 7D).

### 3.7. Structural Comparison of the Active Sites among the 5-FU Bound States of huDHOase, ScDHOase, and PaDHPase

**Figure 8:**
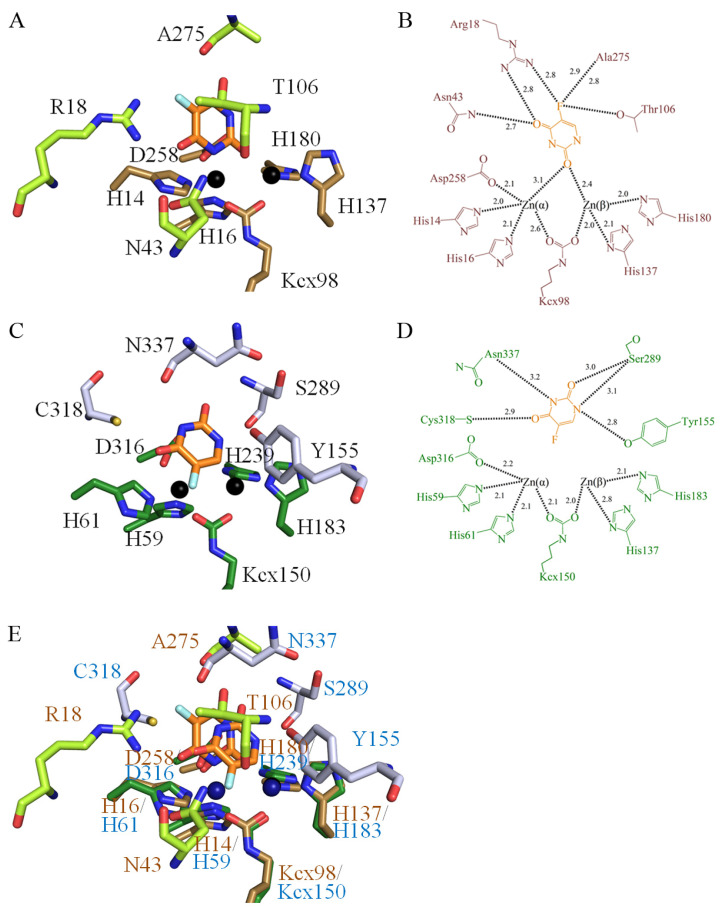
5-FU binding modes. (**A**) Complexed crystal structure of ScDHOase with 5-FU. The two zinc ions in the active site are presented as black spheres. 5-FU is colored in orange. The 5-FU binding sites are colored pale green. The metal binding sites are colored brown. (**B**) The interactions of ScDHOase with 5-FU. R18, N43, T106, and A275 of ScDHOase are involved in 5-FU binding. (**C**) Complexed crystal structure of PaDHPase with 5-FU. The 5-FU binding sites are colored gray. The metal binding sites are colored dark green. (**D**) The interactions of PaDHPase with 5-FU. Y155, C318, S289and N337 of PaDHPase are involved in the 5-FU binding. (**E**) The superimposed structures. Superimposing the 5-FU-complexed structures of ScDHOase (brown) and PaDHPase (blue) indicated the different 5-FU binding modes.

We recently reported the crystal structure of ScDHOase in a complex with 5-FU [28]. Because of the structural resemblance between the active sites of huDHOase (Figure 6A) and ScDHOase (Figure 8A), one might conclude that their 5-FU binding modes must be similar. Indeed, the dynamic loop of ScDHOase extends toward the active site when 5-FU is bound. However, the residues that form their 5-FU binding sites are different. The R18, N43, T106, and A275 of ScDHOase are involved in 5-FU binding (Figure 8B). The corresponding residues in huDHOase are R1475, N1505, F1563, and P1702 (Figure 4). Unlike T106 and A275 in ScDHOase, F1563 and P1702 in huDHOase were too distant from 5-FU to interact with it. Additionally, R1661 and G1703 in huDHOase also interacted with 5-FU through water-molecule-mediated hydrogen bonding. This water molecule does not exist at the active site of ScDHOase. Thus, we concluded that their 5-FU binding mechanisms are different.

The structure of the PaDHPase–5-FU complex is also available for comparison purposes [5]. Aside from huDHOase, PaDHPase is also a member of the cyclic amidohydrolase family [11]. Given that the active sites between huDHOase and PaDHPase are similar (Figure 8C), 5-FU may bind to both of these cyclic amidohydrolases. However, their 5-FU binding modes are significantly different in terms of orientation and binding residues (Figure 8D). Thus, we concluded that the mechanisms that bind 5-FU to huDHOase and PaDHPase are different (Figure 8E).

## 4. Discussion

Metabolic reprogramming allows cancer cells to rapidly proliferate, resist chemotherapies, invade, metastasize, and survive in a nutrient-deprived microenvironment [49]. Many uracil derivatives have long been used as pyrimidine-based antimetabolites for anticancer treatment [4,50]. 5-FU [3] is the best-known fluoropyrimidine drug used to target TSase for anticancer chemotherapy [4]. Over the past 70 years, chemotherapeutic agents that target thymidylate biosynthesis have remained among the most successful drugs used in the treatment of cancer [3,4]. TSase-targeted agents are currently used to treat numerous solid and hematological malignancies, either alone or as foundational therapeutics in combination treatment regimens. However, along with human TSase, many other proteins can also interact with 5-FU. Microbiota can modulate the host response to chemotherapeutic drugs, such as 5-FU [8]. Thus, the whole interactome of 5-FU should be built for detailed clinical pharmacokinetic and toxicity analyses.

In this study, we identified that huDHOase is capable of interacting with 5-FU, with a *K*_d_ value of 91.2 ± 1.7 μM (Figure 7). In order to investigate the binding mode, we solved the complexed crystal structure with 5-FU at a 1.97 Å resolution (Figure 6). The two metal ions and the R1475, N1505, T1562, R1661, D1686, H1690, and G1703 residues were involved in 5-FU binding. This binding mode significantly differs from those of ScDHOase and PaDHPase (Figure 8). In comparison, the *K*_d_ values of 5-FU for ScDHOase and PaDHPase are 192.1 ± 1.4 [28] and 133.2 ± 8.5 μM [5]; thus, the binding abilities of 5-FU follow the order: huDHOase > PaDHPase > ScDHOase. Given that the recommended dose of 5-fluorouracil is >200 mg/m^2^ body surface (or 6 mg/kg) per day, given as continuous intravenous infusion for three weeks [51], these results indicate that, if 5-FU enters into the human body, it prefers to bind to huDHOase over these micro-organism enzymes. Given that the gut microbiome may be different for each person, it is still necessary to determine the binding affinities of 5-FU to any possible protein present in the human body, such as in the gut and blood, for the sake of further comparison and clinical analyses.

Given the similarities in their active sites, the 5-FU binding mode of huDHOase might be considered identical to that of ScDHOase (Figure 8). However, structural analyses revealed that a similar location, but different 5-FU binding poses are found between ScDHOase and huDHOase. Further structural and biochemical experiments are still needed to make the 5-FU binding modes of any protein easier to predict.

**Figure 9:**
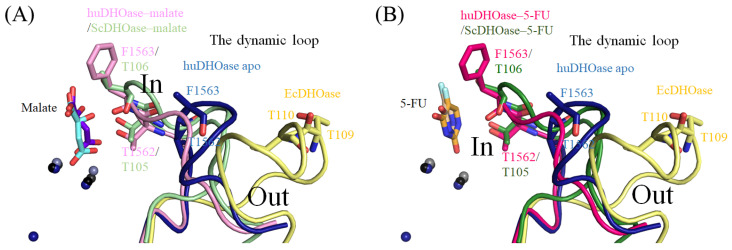
The loop-in binding mode. (**A**) Superposition of the structures of the apo-huDHOase, huDHOase–malate complex, ScDHOase–malate complex, and EcDHOase–5-FOA complex. Three zinc ions in the active site of the apo-huDHOase are presented as dark blue spheres. huDHOase and ScDHOase bound malate via the loop-in mode. When compared, the EcDHOase bound nonsubstrate ligand via the loop-out mode; that is, the loop (yellow) did not interact with the nonsubstrate ligand or with the rest of the active site of EcDHOase. (**B**) Superposition of the structures of apo-huDHOase, the huDHOase–5-FU complex, the ScDHOase–5-FU complex, and the EcDHOase–5-FOA complex. huDHOase and ScDHOase bound 5-FU via the loop-in mode.

Regarding the complexed crystal structures of EcDHOase [29] and huDHOase [22], it is well-established that DHOase binds to the nonsubstrate ligand via the loop-out mode, e.g., the dynamic loop at the active site does not interact with the ligand or with the rest of the active site of DHOase. Namely, the important residues for substrate binding—T109 and T110 in EcDHOase and T1562 and F1563 in huDHOase—should not interact with any nonsubstrate ligand. However, we recently found that ScDHOase binds to malate, a nonsubstrate ligand, via the loop-in mode at pH 6.0, 6.5, 7.0, 7.5, and 9.0, respectively [28,31]. Furthermore, molecular evidence also reveals that ScDHOase binds to 5-FU [28], 5-aminouracil [28], and the inhibitors plumbagin [33] and 5-FOA [32] via the loop-in mode. In order to investigate whether this is a coincidence as a result of being different species, and whether the loop-in binding mode only occurs in simple eukaryotic DHOase (ScDHOase), we determined the crystal structures of huDHOase in a complex with 5-FU and malate. The results of this study showed that the loop in the huDHOase–malate complex (Figure 9A) or the huDHOase–5-FU complex (Figure 9B) is toward the active site. In other words, T1562 and F1563 (Figure 1B) in the catalytic loop of huDHOase are capable of binding to the nonsubstrate ligands. Due to the different experimentally observed phenomena, it may still be too early to draw any conclusions on the common binding modes of DHOases among various species. More complexed structures of DHOase, especially from different species, are still worth determining for a further reproposal of the binding modes.

In this study, we found only two metal ions, rather than the three shown in apo-huDHOase [22], within the active site of these huDHOase complexes (Figure 5 and Figure 6). Biochemically, DHOase, DHPase, and ALLase belong to the cyclic amidohydrolase family and catalyze various hydrolytic reactions at the cyclic amide ring [11]. Almost all of these amidohydrolases, which possess a cluster of four His, an Asp, and a carbamylated Lys, have only two metal ions at their active site. However, the different metal contents of DHOases were still observed. For AaDHOase, only one Zn ion is seen in the active site of each AaDHOase subunit, and the carbamylated Lys is replaced by an Asp [52,53]. EcDHOase [13] and ScDHOase [28] contain two Zn ions at the active site. However, the third Zn ion in apo-huDHOase [22], which was not found in any DHOase, was functionally important for the catalysis of huDHOase. The activity of ScDHOase-T208E, a mutant that has three Zn ions, was enhanced compared with the dimetal enzyme [54]. Thus, whether the binding of malate and 5-FU to the dynamic loop at the active site can cause huDHOase to become a dimetal enzyme still needs further investigation.

The crystal structures of HYDase [55], DHPase [56], DHOase [13,57], and ALLase [58] reveal that the chemical mechanism of these binuclear-metal-center-containing cyclic amidohydrolases likely consists of three main steps [11]: (I) the hydrolytic water molecule must be activated to enable a nucleophilic attack; (II) the amide bond of the substrate must be made to be electrophilic by the polarization of the carbonyl O bond, (III) and the leaving group N must be protonated as the C–N bond is cleaved. The flexible loop in DHOase is also crucial for stabilizing the transition state, supporting the movement of this loop being part of the catalytic cycle [29]. In addition to the binding of the substrate [29], our structural evidence further indicated that this loop in huDHOase is also involved in the binding of the inhibitor malate. A similar dynamic loop can also be found in DHPase [11]. Regardless of their different sequences and the binding modes, the flexible loop in DHPase [11,59] was crucial for the catalysis. Thus, the dynamic active site loop in DHOase and DHPase should be suitable drug targeting sites for selectively inhibiting pyrimidine metabolism [59,60].

## 5. Conclusions

In this study, we identified that huDHOase is a novel 5-FU binding protein, thereby extending the 5-FU interactome to this human enzyme. Mutational and structural analyses indicated that the 5-FU binding mode of huDHOase differed from ScDHOase and PaDHPase. Considering the loop-in mode revealed by our structures complexed with huDHOase, the dynamic loop in DHOase should be a suitable drug target for inhibiting pyrimidine biosynthesis. This complex structure might provide insights into how 5-FU and its pyrimidine derivatives could bind to and inhibit the proteins in cancer-signaling pathways. We also found a potential interface for the dimerization of huDHOase. The subunit-interacting residues of huDHOase for dimerization are significantly different from other DHOases. Given that microbiota can modulate the host response to 5-FU, further research should directly focus on revisiting the role of bacterial and human DHOase in anticancer therapy.

## Figures and Tables

**Table 1:** Data collection and refinement statistics.

Data Collection		
Crystal	huDHOase–Malate	huDHOase–5-FU
Wavelength (Å)	1.00	1.00
Resolution (Å)	30–1.64	30–1.97
Space group	C222_1_	C222_1_
Cell dimension (Å)	a = 82.05 α = 90°	a = 81.78 α = 90°
	b = 158.25 β = 90°	b = 157.79 β = 90°
	c = 61.19 γ = 90°	c = 61.83 γ = 90°
Completeness (%)	98.9 (98.6) *	99.9 (99.8) *
<I/σ_I_>	11.51 (2.41)	29.84 (6.63)
CC_1/2_	0.998 (0.807)	0.991 (0.972)
Redundancy	7.0 (7.0)	7.0 (7.2)
Refinement		
Resolution (Å)	28.53–1.64	28.78–1.97
No. reflections	48510	28310
*R*_work_/*R*_free_	0.172/0.191	0.174/0.214
No. atoms		
Protein	3072	3063
Ligand	15	14
Zinc	2	2
Water	269	260
R.m.s deviation		
Bond lengths (Å)	0.006	0.008
Bond angles (°)	0.951	1.012
Ramachandran plot		
In preferred regions	96.36%	96.01%
In allowed regions	3.08%	3.43%
Outliers	0.56%	0.56%
PDB entry	8GW0	8GVZ

* Values in parentheses are for the highest resolution shell. CC_1/2_ is the percentage of correlation between the intensities of random half-data sets.

**Table 2:** The formation of hydrogen bonds at the monomer–monomer interface of the huDHOase dimer A–A′ complexed with malate or 5-FU.

Subunit A	Subunit A′	Distance [Å] (the Malate Complex)	Distance [Å] (the 5-FU Complex)
T1595 [OG1]	Q1594 [OE1]	2.4	2.4
R1630 [NE]	L1605 [O]	2.7	3.0
R1630 [NH2]	L1605 [O]	2.8	3.1
R1630 [NH2]	Q1607 [OE1]	2.4	2.5
V1571 [N]	E1619 [OE2]	2.8	2.8
Q1594 [OE1]	T1595 [OG1]	2.4	2.4
L1605 [O]	R1630 [NE]	2.7	3.0
L1605 [O]	R1630 [NH2]	2.8	3.1
Q1607 [OE1]	R1630 [NH2]	2.4	2.5
E1619 [OE2]	V1571 [N]	2.8	2.8

The formation of hydrogen bonds at the monomer–monomer interface of the complex was found by using PISA (Protein Interfaces, Surfaces, and Assemblies) analysis [46].

**Table 3:** Binding parameters of DHOases.

Protein	*λ*_max_ (nm)	*λ*_em_ Shift (nm)	Quenching (%)	*K*_d_ Value (µM)
huDHOase	From 340 to 349	8.5	94.5	91.2 ± 1.7
huDHOase-T1562A	From 340 to 346	6.0	89.0	146.5 ± 2.1
huDHOase-R1475A	From 339.5 to 341.5	2.0	75.3	161.5 ± 1.6

**Table 4:** Primers used for construction of plasmids.

Oligonucleotide	Primer
huDHOase-R1475A-N	TCCATGTGCACCTGGCGGAACCAGGTGGGA
huDHOase-R1475A-C	CACCTGGTTCCGCCAGGTGCACATGGACAT
huDHOase-T1562A-N	CTTTACCTCAATGAGGCCTTCTCTGAGCTG
huDHOase-T1562A-C	GCCGCAGCTCAGAGAAGGCCTCATTGAGGT

Underlined nucleotides indicate the designated site for mutation site.

## Data Availability

Atomic coordinates and related structure factors were deposited in the PDB with accession codes 8GW0 and 8GVZ.
